# Magnetically Aligned and Enriched Pathways of Zeolitic Imidazolate Framework 8 in Matrimid Mixed Matrix Membranes for Enhanced CO_2_ Permeability

**DOI:** 10.3390/membranes10070155

**Published:** 2020-07-17

**Authors:** Machiel van Essen, Esther Montrée, Menno Houben, Zandrie Borneman, Kitty Nijmeijer

**Affiliations:** Membrane Materials and Processes, Department of Chemical Engineering and Chemistry, Eindhoven University of Technology, P.O. Box 513, 5600 MB Eindhoven, The Netherlands; m.v.essen@tue.nl (M.v.E.); e.t.j.montree@gmail.com (E.M.); m.houben@tue.nl (M.H.); z.borneman@tue.nl (Z.B.)

**Keywords:** mixed matrix membranes, zeolitic imidazolate frameworks, alignment, CO_2_ based separations

## Abstract

Metal-organic frameworks (MOFs) as additives in mixed matrix membranes (MMMs) for gas separation have gained significant attention over the past decades. Many design parameters have been investigated for MOF based MMMs, but the spatial distribution of the MOF throughout MMMs lacks investigation. Therefore, magnetically aligned and enriched pathways of zeolitic imidazolate framework 8 (ZIF−8) in Matrimid MMMs were synthesized and investigated by means of their N_2_ and CO_2_ permeability. Magnetic ZIF−8 (m–ZIF−8) was synthesized by incorporating Fe_3_O_4_ in the ZIF−8 structure. The presence of Fe_3_O_4_ in m–ZIF−8 showed a decrease in surface area and N_2_ and CO_2_ uptake, with respect to pure ZIF−8. Alignment of m–ZIF−8 in Matrimid showed the presence of enriched pathways of m–ZIF−8 through the MMMs. At 10 wt.% m–ZIF−8 incorporation, no effect of alignment was observed for the N_2_ and CO_2_ permeability, which was ascribed anon-ideal tortuous alignment. However, alignment of 20 wt.% m–ZIF−8 in Matrimid showed to increase the CO_2_ diffusivity and permeability (19%) at 7 bar, while no loss in ideal selectivity was observed, with respect to homogeneously dispersed m–ZIF−8 membranes. Thus, the alignment of MOF particles throughout the matrix was shown to enhance the CO_2_ permeability at a certain weight content of MOF.

## 1. Introduction

Polymeric membranes for gas separation are used for purification, generation and separation of gaseous streams, besides other methods, such as (cryogenic) distillation, pressure swing adsorption and amine scrubbing [1]. In comparison with these other technologies, gas separation membranes may simplify the operational process, provide a relatively easy possibility for upscaling and require no phase change [1]. Despite these advantages, polymeric membranes exhibit a negative correlation between selectivity and permeability, which is known as Robeson’s upper bound [2,3]. The incorporation of additives in polymeric membranes, i.e., a mixed matrix membrane (MMM), can result in shifting the membrane performance towards this upper bound or even surpassing it [4,5,6,7]. Additives that are used to enhance the MMM separation performance and have gained significant attention over the past decades are metal-organic frameworks (MOFs) [8]. MOFs are microporous crystalline networks where metal ions, i.e., the nodes, are coordinated by organic linkers. Like other microporous materials, such as zeolites, MOFs have large surface areas. The chemical affinity, surface area and pore size of MOFs are highly tunable by varying the linker between the metal nodes. These exceptional properties make MOFs promising candidates for the application in the field of gas separation and storage [9]. 

Zeolitic imidazolate frameworks (ZIFs) are a subclass of MOFs with a crystal structure that exists out of zinc or cobalt cations and imidalozate linkers [10]. ZIFs owe their name to the metal-imidazolate-metal bond angle, which is the same as the Si-O-Si bond angle in zeolites. As the chemical and thermal stability can be an issue in the application of MOFs, ZIFs are a promising subclass of MOFs due to their outstanding chemical and thermal stability [11,12]. One particular type of ZIF that has gained significant attention in the field of gas separation MMMs is ZIF−8, which exists out of zinc ions and 2-methylimidazolate (mIm) linkers [10]. Due to the pore dimensions (pore aperture = 3.4 Å, pore diameter = 11.6 Å), ZIF−8 exhibits high gas uptake and sieving properties towards gases with a small kinetic diameter, e.g., CO_2_ and H_2_ [10,13]. The size and shape of ZIF−8, which has an isotropic sodalite topology, can be tuned by a variety of parameters, such as solvent type, zinc salt type, precursor ratio, concentrations of the precursor and synthesis method [10,14,15,16,17,18]. Besides the size and shape of ZIF−8, catalytic and magnetic properties can be introduced to the MOF by adding nanoparticles to the synthesis mixture, which extends the field of application of ZIF−8 [17,19,20,21]. Pure ZIF−8 membranes on porous supports and ZIF−8 based MMMs have demonstrated the potential of ZIF−8 for gas separation applications, by means of relatively high selectivity and permeability [8,13,22,23,24]. Even membranes of single crystal ZIF−8 structures have been reported [25]. Generally, the addition of ZIF−8 in a polymer matrix, to form MMMs, results in an increased gas permeability. Moreover, depending on the gas pair and MOF load, the selectivity can improve as well [8].

MMMs consisting of a polymeric matrix with MOF additives provide a wide field of research, where the MMM can be optimized in various manners, since there are many parameters to tune. Firstly, by introducing CO_2_-philic linkers in MOFs, the separation of CO_2_/CH_4_ by MMMs can be significantly improved [5]. Interfacial compatibility between the matrix and the MOF can be optimized by matrix modification or the addition of a dispersant, where the improved MOF embedding results in increased CO_2_/CH_4_ selectivity [26,27]. The MOF size is an important factor as well for the MMM performance. For example, increasing the MOF size from 50 to 150 nm results in improved H_2_/CO_2_ selectivity and permeability. This observation is ascribed to the decreased particle agglomeration in the matrix due to the decreased external surface area of the MOF particles [28]. Lastly, annealing temperature of MMMs affects their performance as well. Both the mechanical properties (e.g., toughness, hardness, yield and fracture strength), as well as the permeability of MMMs, show optimal performance when annealed at elevated temperatures [29,30].

One type of optimization of MOF based MMMs that is only marginally explored in the field of gas separation membranes is the spatial distribution of MOFs in MMMs, i.e., aligning three-dimensional isotropic MOFs parallel to the direction of permeation through the membrane. This MOF alignment should form “molecular highways” through the membrane due to the highly permeable nature of MOFs, with respect to the polymer matrix. Alignment of one-dimensional and two-dimensional additives has been performed in MMMs. Techniques for achieving these aligned MMMs were based on external magnetic and electric fields or substrate directed, i.e., a template determines the orientation [31,32,33,34]. For both one-dimensional (i.e., carbon nanotubes) and two-dimensional (i.e., montmorillonite) additives, an improved gas separation performance was observed [32,33,34]. Both one and two-dimensional alignment depends on the orientation of the additive, e.g., carbon nanotubes (a one-dimensional additive), which are permeable through their interior, are oriented parallel to the permeation path. However, three-dimensional additives, e.g., ZIF−8, are permeable in the x, y and z direction, due to their isotropy. Therefore, alignment of three-dimensional additives does not rely on spatial orientation, but on spatial rearrangement. 

Hypothetically, the permeability of MMMs with isotropic MOFs should be enhanced by alignment of the MOF, if the permeability of the MOF is significantly higher than the matrix. Alignment will form pathways of the three-dimensional MOF and as a consequence will the permeation behavior be shifted towards the MOF permeability. Thus, the distance between MOFs will be reduced due to the alignment. The permeation through the matrix, i.e., the rate determining component, is minimized through these permeation paths. This improved use of the MOFs could hypothetically reduce the amount of MOFs required in MMMs to obtain a desired permeability. 

Therefore, this research aims to align a three-dimensional isotropic additive in an MMM in such a manner that the MOF will form local clusters which will lie parallel to the gas permeation path. Since it has been shown that magnetic properties can be introduced in ZIF−8 and that ZIF−8 as an additive in MMMs increases permeability, this MOF is selected as additive for the MMM. The polyimide Matrimid^®^ 5218 is selected as polymer matrix. Nanoscale sized magnetite (Fe_3_O_4_), an iron oxide with a significant higher magnetic susceptibility than other iron oxides, will be used as ferrimagnetic material to magnetize ZIF−8. Additive alignment will be done in a magnetic field, since other magnetic isotropic particles have already been successfully aligned in the direction of the magnetic field lines by magnetic fields in composite materials [35,36]. Lastly, the MMMs will be evaluated for their single gas permeability of CO_2_ and N_2_. This gas pair is selected since it may resemble partially a flue gas composition [37]. Moreover, N_2_ behaves similar to CO by means of thermal diffusion [38]. Furthermore, the kinetic diameters and critical temperatures both gases are in the same range (d_N_2__ = 3.64 Å, d_CO_ = 3.76 Å, T_c,N_2__ = −147 °C, T_c,CO_ = −140 °C). Therefore, CO_2_/N_2_ gas mixtures resemble partially the output stream of CO_2_ plasma conversion into CO as well [39].

## 2. Materials and Methods 

### 2.1. Chemicals

Magnetite (Fe_3_O_4_, d_average_ = 8 nm, 99.9% purity, US Research Nanomaterials, Houston, TX, USA), 2-methylimidazole (mIm, 99% purity, Sigma-Aldrich, Zwijndrecht, The Netherlands), poly(ethylene glycol) (PEG, 1 kDa, Sigma-Aldrich, Zwijndrecht, The Netherlands), N,N-dimethylformamide (DMF, ≥99.9% purity, Sigma-Aldrich, Zwijndrecht, The Netherlands), zinc nitrate hexahydrate (Zn(NO_3_)_2_ · 6 H_2_O, 98% purity, Thermofischer Acros Organic, Geel, Belgium), Matrimid^®^ 5218 (Matrimid, kindly provided by Huntsman, Basel, Switzerland), ultrapure water (PURELAB Option demineralizer, Veolia Water Solutions and Technologies, Ede, The Netherlands), helium (5.0 grade, Linde gas, Schiedam, The Netherlands), nitrogen (4.5 grade, Linde gas, Schiedam, The Netherlands) and carbon dioxide (4.5 grade, Linde gas, Schiedam, The Netherlands) were used as received without further purification.

### 2.2. Magnetic ZIF−8 Synthesis

Magnetic ZIF−8 (m–ZIF−8) and ZIF−8 were prepared by an aqueous room temperature method. First, 10 mL ultrapure water was added to 20 mg Fe_3_O_4_. The suspension was sonicated with a Sonics Vibra-Cell ultrasonication probe (Sonics and Materials Inc., Newtown, Connecticut, USA) at 35% probe intensity for 10 min. Meanwhile, 500 mg PEG, which is used as surfactant, was dissolved in 10 mL ultrapure water. The sonicated Fe_3_O_4_ suspension and PEG solution were added together and the mixture was stabilized for 15 min. Again, the mixture was sonicated with a sonication probe for 10 min and stabilized for 15 min. The PEG stabilized Fe_3_O_4_ was washed three times with ultrapure water to remove the excess PEG by simply containing the Fe_3_O_4_ with a magnet. 10 mL of ultrapure water was added to the magnetic particles. 150 mg Zn(NO_3_)_2_ · 6 H_2_O (0.50 mmol) was added to the stabilized Fe_3_O_4_ suspension. A solution of 1.64 g mIm (20 mmol) in 15 mL ultrapure water was added to the mixture. The mixture was stirred for 30 min while ZIF−8 formation occurred. The product was washed three times by containing the product with a magnet. M-ZIF−8 was dried in a vacuum oven overnight at 75 °C and 350 mbar. Lastly, the product was further dried for 24 hr at 100 °C and 0 mbar. Traditional ZIF−8 was synthesized as a reference by the same method as the m–ZIF−8 synthesis, but without the presence of PEG stabilized Fe_3_O_4_. Since ZIF−8 is not magnetic by nature, washing was performed by centrifuging the ZIF−8 suspension with a CompactStar CS 4 (VWR, Amsterdam, The Netherlands) for 20 min at 6500 rpm three times.

### 2.3. Membrane Formation

#### 2.3.1. Native Matrimid Membranes

A total of 5 g Matrimid was dissolved in 20 g DMF overnight, resulting in a 20 wt.% total solids polymer solution. This solution was cast on a glass plate with a 0.5 mm casting knife. Evaporation of the DMF in a nitrogen box for two days solidifies the polymer membrane. To completely dry the cast flat sheet membrane, the membrane was put in a nitrogen oven at 80 °C for two days and subsequently in a vacuum oven at 125 °C and 0 mbar for two days.

#### 2.3.2. Homogeneous m–ZIF−8/Matrimid MMMs

The required m–ZIF−8 amount was added to 20.5 g DMF (0.45 g for 10 wt.%, 0.90 g for 20 wt.%). The suspension was sonicated in a Branson 3510 sonication bath for 30 min. Matrimid was added to the MOF suspension in three stages (4.05 g in total for 10 wt.% and 3.6 g in total for 20 wt.%), which results in casting solutions with 18 wt.% total solids. The MMM solutions were stirred overnight. MMMs were cast from these solutions on a glass plate with a 0.5 mm casting knife. The obtained homogeneous MMMs were dried in the same manner as the pure Matrimid membranes. The obtained 10 and 20 wt.% homogeneous (H) m–ZIF−8/Matrimid MMMs are abbreviated to 10 wt.% H and 20 wt.% H, respectively.

#### 2.3.3. Magnetically Aligned m–ZIF−8/Matrimid MMMs

**Figure 1:**
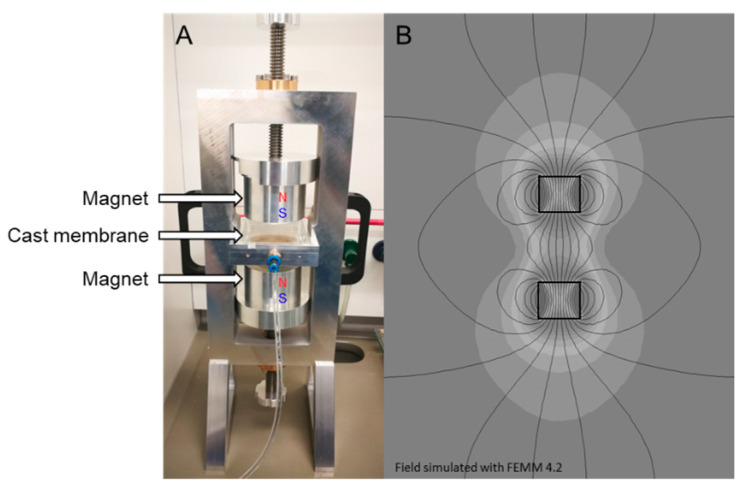
(**A**) The magnetic alignment setup. The nitrogen box is placed in the middle of two magnets from which both their magnetic north pole is faced upwards. (**B**) Simulation of the obtained non-homogeneous magnetic field lines with a gap of 80 mm between the magnets.

Magnetically aligned m–ZIF−8/Matrimid MMMs were made similarly to their homogeneous counterpart, where the only difference is the first day of the solvent evaporation. For the homogenous MMM preparation, solvent evaporation in a nitrogen box from the cast polymer solution forms the membrane, whereas for the preparation of the aligned MMMs the nitrogen box was placed in the middle of a permanent magnetic field for one day (Figure 1A). To prevent excessive m–ZIF−8 migration, an aluminum ring (d = 25 mm) was placed in the center of the alignment setup. After one day of solidification in the magnetic field, the membrane was placed in a normal nitrogen box and the previously mentioned drying procedure was followed. This approach of the MOF alignment in MMMs should also be suitable for extension to other types of MOFs and matrices. However, if the MOF contains a one-dimensional pore system, other strategies, such as exterior decoration with Fe_3_O_4_, should be considered, as the incorporation of Fe_3_O_4_ in a one-dimensional system might result in pore blocking. The obtained 10 and 20 wt.% aligned m–ZIF−8/Matrimid MMMs are abbreviated to 10 wt.% Al and 20 wt.% Al, respectively. Higher weight percentages of m–ZIF−8/Matrimid were also attempted to be aligned, but because of the high M–ZIF−8 volume fraction this did not result in defect free membranes.

The magnetic field was created by two N45 grade permanent magnets (d = 70 mm, h = 60 mm, Nb-Fe-B axially magnetized). The magnetic field strength in the middle of the magnets was 0.22 T when the gap between the magnets was set to 80 mm. The authors are aware that the obtained magnetic field is non-homogeneous, i.e., the field strength decreases from the middle point towards the outer parts of the setup (Figure 1B). However, experiments were also performed in homogeneous magnetic fields created by an electromagnet, but the heat generated by the coils of the electromagnet interfered with the solidification process.

### 2.4. Characterization

The crystal structure of Fe_3_O_4_, ZIF−8 and m–ZIF−8 was analyzed by X-ray diffraction (XRD) using a Rigaku Miniflex 600 (Wilmington, MA, USA) (15 mA, 40 kV, Cu Kα radiation (λ = 1.5406 Å)) in the 2 θ range from 2° to 80° with a scanning rate of 0.5°/min. 

The Brunauer–Emmett–Teller (BET) surface area of Fe_3_O_4_, ZIF−8 and m–ZIF−8 was determined by N_2_ physisorption with a Micromeretics Tristar II (Norcross, GA, USA) at −196 °C (liquid N_2_). Prior to the measurement, the samples were dried in a vacuum oven at 100 °C and 0 mbar overnight. 

The thermal stability of Fe_3_O_4_, ZIF−8, m–ZIF−8, Matrimid and the MMMs was investigated with a PerkinElmer (Waltham, MA, USA) TGA 4000 under N_2_ atmosphere (flow 20 mL/min) from 50 to 800 °C with a heating rate of 20 °C/min.

The external morphology of ZIF−8, m–ZIF−8 and the cross-sections of the MMMs were analyzed by a scanning electron microscope (SEM, JEOL JSM-IT100, Tokyo, Japan). Cross-sections of MMMs were obtained by immersing the membranes in a 50:50 water/isopropanol mixture and subsequently fracturing them after immersion in liquid N_2_. Prior to the analysis, all samples were sputter coated with a JEOL JFC−2300 HR (Tokyo, Japan) with platinum source. 

The elemental composition and dispersion of ZIF−8, m–ZIF−8 and the MMMs were analyzed with energy dispersive X-ray spectroscopy (EDS, JEOL JSM-IT100, Tokyo, Japan). Samples were prepared in the same manner as the SEM analysis.

High pressure gas sorption of N_2_ and CO_2_ was performed at 35 °C for ZIF−8, m–ZIF−8, Matrimid and the MMMs (Rubotherm series IsoSORP^®^ sorption instrument, Bochum, Germany). The equipment measures the sorbed weight of the gas with a magnetically suspended balance. Prior to each sorption measurement, the weight and volume of the sample is determined with a helium reference measurement. With the obtained sample weight and volume, the measured sorbed weight is corrected for the buoyancy of the measurement gases according to Equation (1), where m_corrected_ is the corrected weight (g), m_measured_ the measured weight (g), ρ_gas_ the density of the measuring gas (g/cm^3^) and V_sample_ the sample volume (cm^3^).

(1) $$m_{\mathrm{corrected}}{=m}_{\mathrm{measured}}{+\rho }_{\mathrm{gas}}\cdot V_{\mathrm{sample}}$$

The single gas permeability of Matrimid and the MMMs for N_2_ and CO_2_ was determined at 35 °C in triplo by measuring permeate pressure increase over time in a calibrated volume at pressures ranging from 7 to 27 bar according to Equation (2). In Equation (2), P_i_ is the permeability of gas species i (Barrer), Δp_permeate_ the increase in permeate pressure (Pa) per time interval Δt (s), V_c_ the calibrated permeate volume (m^3^), R the gas constant (J/(K∙mol)), T the permeate temperature (K), V_m_ the molar volume at STP (cm^3^/mol), L the membrane thickness (cm), A the membrane area (cm^2^) and Δp the transmembrane pressure (cmHg).

(2) $$P_{i}=\frac{{\Delta p}_{\mathrm{permeate}}\cdot V_{c}\cdot V_{m}\cdot L\cdot 10^{10}}{\Delta t\cdot R\cdot T\cdot A\cdot \Delta p}$$

Prior to each single gas permeation measurement, the membranes were first conditioned for 8 hr at 35 °C and 2 bar N_2_. Hereafter, first, the N_2_ single gas permeation measurements were performed. Then, the CO_2_ single gas permeation measurements were performed, since the CO_2_ measurements plasticize the membranes. The ideal CO_2_/N_2_ selectivity, α_CO_2_/N_2__ (−), was calculated according to Equation (3).

(3) $$\alpha_{{\mathrm{CO}}_{2}{/N}_{2}}{=P}_{{\mathrm{CO}}_{2}}{/P}_{N_{2}}$$

The CO_2_ diffusion coefficient of Matrimid and the MMMs was determined according to Equation (4), where D is the CO_2_ diffusion coefficient (cm^2^/s), P the CO_2_ permeability (Barrer) and S the CO_2_ solubility coefficient (cm^3^ STP/(cm^3^·cmHg)). The permeability was experimentally determined by permeation experiments whereas the CO_2_ solubility coefficient was experimentally obtained by the high pressure sorption measurements using a magnetic suspension balance.

(4) D=P/S

## 3. Results and Discussion

### 3.1. ZIF−8, m–ZIF−8 and Fe_3_O_4_ Analysis

**Figure 2:**
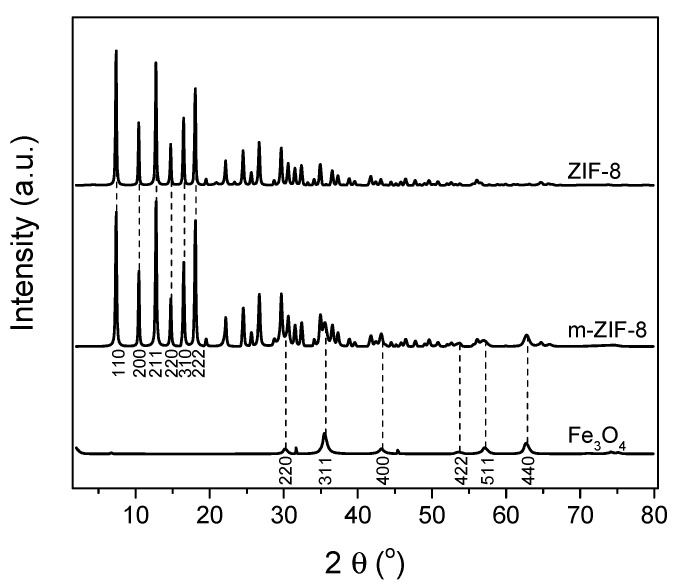
XRD patterns of ZIF−8, m–ZIF−8 and Fe_3_O_4_.

The XRD patterns (Figure 2) show the typical diffraction peaks for both Fe_3_O_4_ and ZIF−8. The typical diffraction peaks of both materials are in agreement with the crystal structures found in literature [16,40]. It is clear that the diffraction pattern of m–ZIF−8 shows both the diffraction peaks of ZIF−8 and Fe_3_O_4_, which means that the crystal structures of both particles are present in the composite m–ZIF−8 crystal. The ratio of the (211) ZIF−8 peak and the (440) Fe_3_O_4_ peak in m–ZIF−8 is higher than the ratio found in literature for ZIF−8/Fe_3_O_4_ composites where a significant lower relative amount of Fe_3_O_4_ is used in the synthesis mixture [19,21]. On the other hand, the peak ratio of m–ZIF−8 is lower than the ratio found in literature for ZIF/Fe_3_O_4_ composites, where the Fe_3_O_4_ particle size was significantly bigger [20]. This observation indicates that a considerable amount of Fe_3_O_4_ nanoparticles is present in the m–ZIF−8 crystals. 

**Figure 3:**
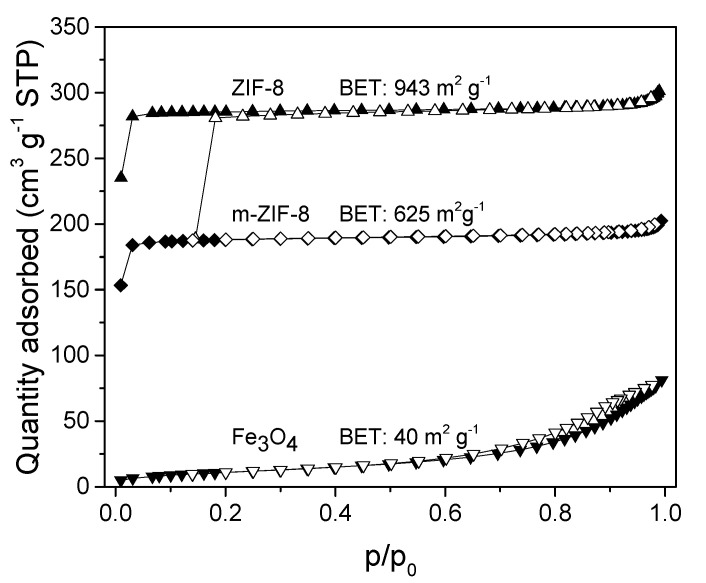
N_2_ adsorption isotherms (filled = adsorption, open = desorption) and BET surface areas of ZIF−8, m–ZIF−8 and Fe_3_O_4_.

The influence of Fe_3_O_4_ incorporation in ZIF−8 on the N_2_ adsorption is visible in Figure 3. The BET surface area of 943 m^2^/g of ZIF−8 is comparable to BET surfaces areas reported in literature for ZIF−8 aqueous syntheses at room temperature and confirms the presence of permanent microporous system [14,41]. The BET surface area of m–ZIF−8 (625 m^2^/g) is clearly lowered by the presence of non-microporous Fe_3_O_4_ (40 m^2^/g) in comparison with pure ZIF−8, i.e., a decrease of 33.7%. ZIF−8/Fe_3_O_4_ composites with relatively lower amounts of Fe_3_O_4_ in the synthesis mixture show less decrease in surface area [21]. 

**Figure 4:**
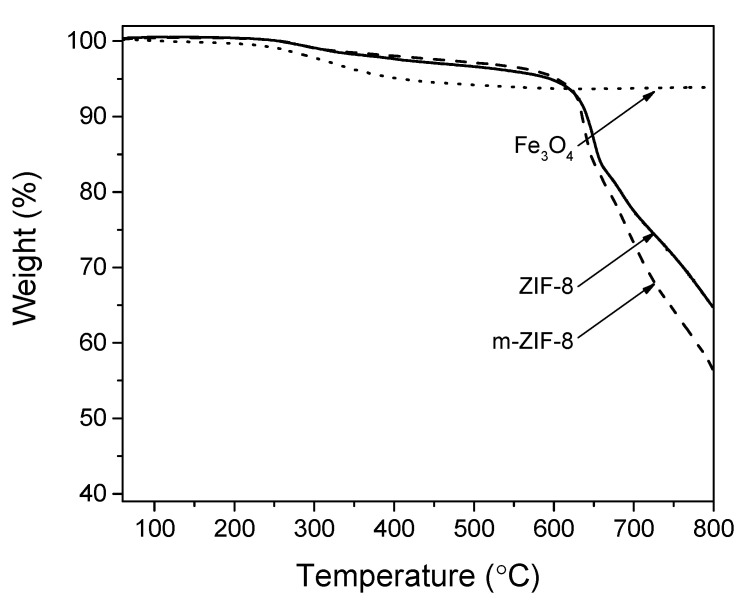
Thermal stability of ZIF−8, m–ZIF−8 and Fe_3_O_4_.

ZIF−8, m–ZIF−8 and Fe_3_O_4_ show excellent thermal stability (Figure 4). Incorporation of highly stable Fe_3_O_4_ does not affect the degradation temperature of m–ZIF−8 in comparison with ZIF−8. Both ZIF−8 and m–ZIF−8 show a degradation temperature of approximately 600 °C. The small weight loss of ZIF−8 and m–ZIF−8 from 250 to 600 °C is attributed to partial carbonization of entrapped guest molecules, e.g., residual 2-methylimidazole [10].

**Figure 5:**
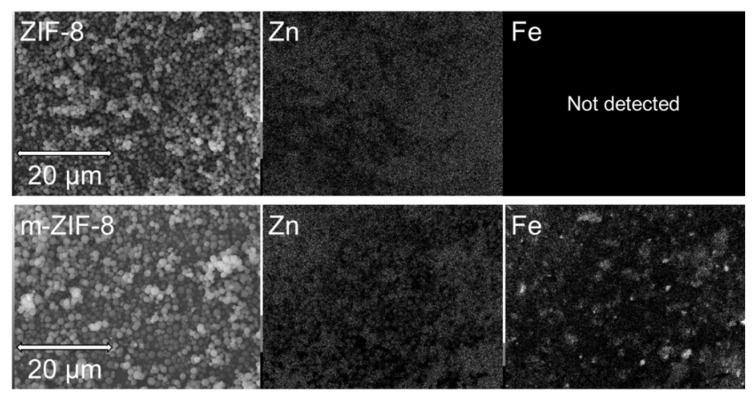
SEM images of ZIF−8 and m–ZIF−8 and their corresponding EDS maps of Zn and Fe.

SEM/EDS analysis of the morphology and elemental composition of ZIF−8 and m–ZIF−8 shows two clear differences between the reference and the magnetized ZIF−8 samples (Figure 5). First, the particle size and range of particle size is increased when Fe_3_O_4_ is added to the synthesis mixture of ZIF−8. The ZIF−8 particle size from ranges 0.6 to 1 µm and the m–ZIF−8 particle size ranges from 1 to 1.6 µm. Apparently, the presence Fe_3_O_4_ in the m–ZIF−8 synthesis acts as nucleation point, which causes a slightly increased particle size. Secondly, and obviously, no Fe is detected by EDS for ZIF−8 and Fe is detected for the m–ZIF−8. 

**Figure 6:**
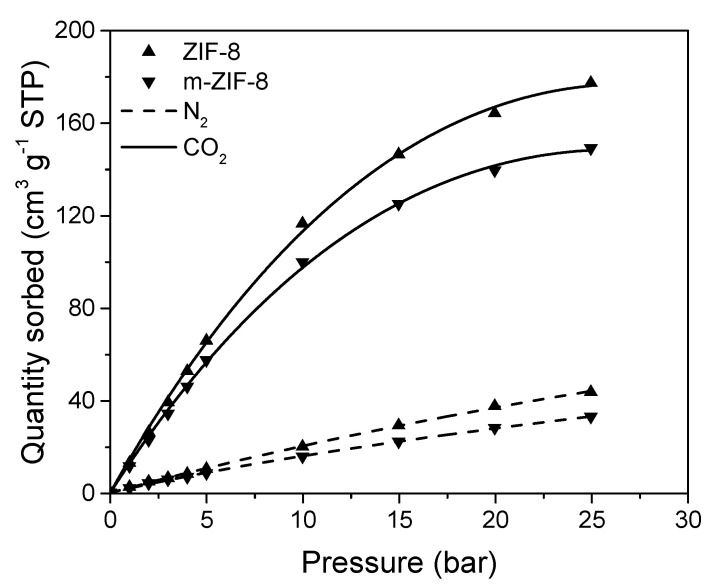
N_2_ (dash) and CO_2_ (solid) sorption isotherms of ZIF−8 (triangle up) and m–ZIF−8 (triangle down).

The effect of the incorporation of Fe_3_O_4_ on the N_2_ and CO_2_ sorption in m–ZIF−8 is shown in Figure 6 and confirms the observation made for the decrease in BET surface area of m–ZIF−8. Since Fe_3_O_4_ has no microporosity, a decrease in both N_2_ and CO_2_ sorption is observed when Fe_3_O_4_ is incorporated in ZIF−8. The sorption of N_2_ in ZIF−8 and m–ZIF−8 shows Henry sorption behavior, since almost linear sorption behavior is observed. On the other hand, Langmuir sorption behavior is observed for CO_2_ in both ZIF types, since the slope of sorption decreases over the whole pressure range. Clearly, higher quantities of CO_2_ are sorbed in both ZIF structures than N_2_. This indicates that the sorption of CO_2_ is thermodynamically favored over N_2_ sorption, which was also observed for another ZIF type [42]. Moreover, the observed sorption ratio of the two gases corresponds with simulated ratios of sorption of ZIF−8 at 25 °C [43]. 

### 3.2. Membrane Analysis

**Figure 7:**
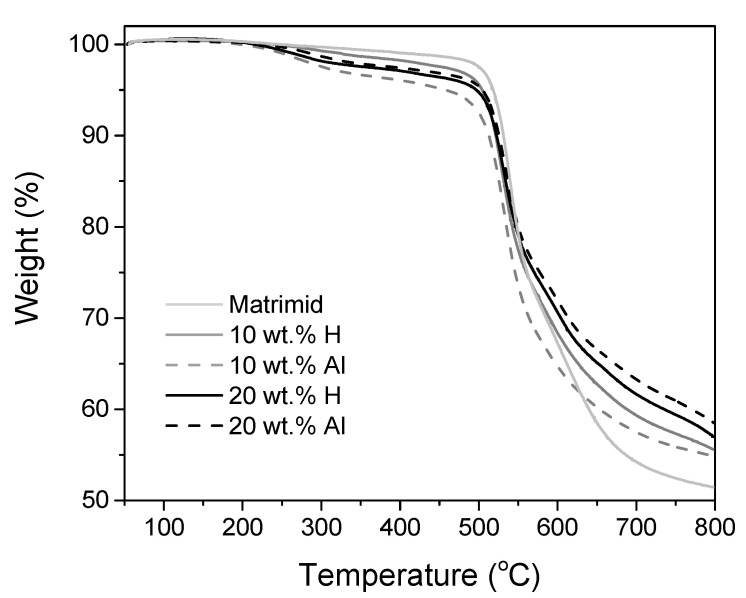
Thermal stability of Matrimid, 10 wt.% H, 10 wt.% Al, 20 wt.% H and 20 wt.% Al MMMs.

TGA confirms that the drying method was sufficient for Matrimid and the MMMs (Figure 7). The membranes contained no solvent, since no weight loss observed in the trajectory from approximately 150 to 200 °C (boiling point DMF = 153 °C). All membranes have a degradation temperature of approximately 500 °C. The degradation temperature of m–ZIF−8 (600 °C, Figure 4) is 100 °C higher in comparison with Matrimid that has a degradation temperature of 500 °C (Figure 7). Since the MMMs contain at least 80 wt.% Matrimid, the difference between pure Matrimid and the MMMs is marginally visible.

**Figure 8:**
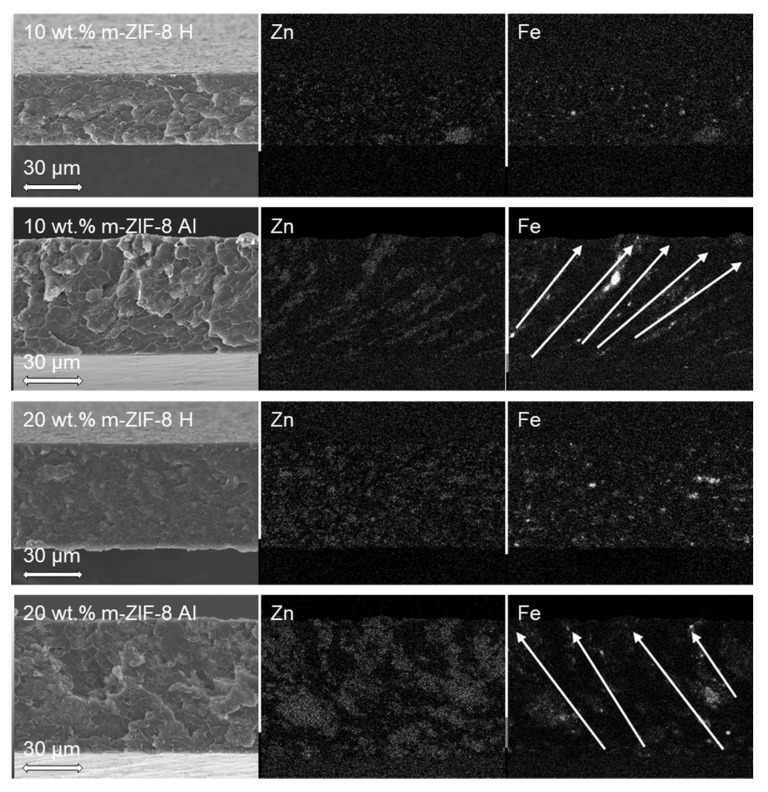
SEM images of 10 wt.% H, 10 wt.% Al, 20 wt.% H and 20 wt.% Al MMMs and their corresponding EDS maps of Zn and Fe, the arrows indicate the direction of alignment. All membranes have glass plate side at the bottom side of the SEM image and EDS mapping.

The morphology and elemental distribution of cross-sections of the homogeneous and aligned MMMs are shown in Figure 8.

Both the 10 wt.% H and 20 wt.% H MMMs show that m–ZIF−8 is evenly distributed throughout the matrix, i.e., the characteristic elements Zn and Fe are evenly distributed throughout both the membranes. On the other hand, both the 10 wt.% Al and 20 wt.% Al MMMs show that lines and enriched regions of Zn and Fe are present through the matrix. This confirms that m–ZIF−8 is effectively clustered and aligned in the magnetic field lines (indicated by the arrows in Figure 8). Compact lines of Zn and Fe are present in the 10 wt.% Al MMM, which partially reach from the bottom to the top of the membrane. The 20 wt.% Al MMM shows that increasing the amount of incorporated m–ZIF−8 results in broader aligned pathways in comparison with the 10 wt.% Al MMM. Thus, increasing the additive content during alignment results in a further increased interconnectivity between m–ZIF−8 particles. Similar morphologies and Fe mappings are reported for other composites with magnetically aligned particles [35,36]. Alignment of m–ZIF−8 increases thickness of the MMMs, since the m–ZIF−8 particles are attracted towards the magnetic field lines. The thickness increases with 24 µm for the 10 wt.% MMMs (L_10 wt.% H_ = 36 µm, L_10 wt.% Al_ = 60 µm) and with 16 µm for the 20 wt.% MMMs (L_20 wt.% H_ = 54 µm, L_20 wt.% Al_ = 70 µm). It is visible for the aligned MMMs that the used magnetic field is inhomogeneous, since alignment of the MMMs is not perpendicular to the membrane surface, which increases the MOF path length through the matrix.

**Figure 9:**
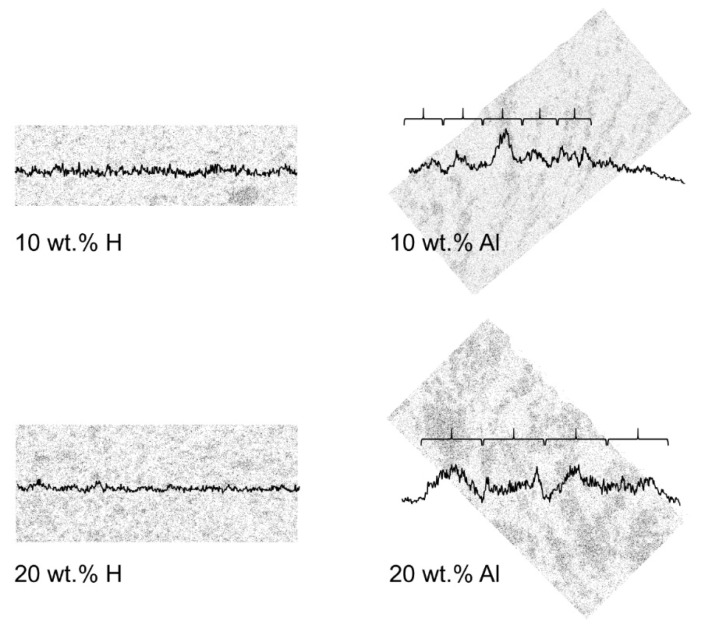
Particle count of Zn EDS maps (color inversed with respect to Figure 8) of 10 wt.% H, 10 wt.% Al, 20 wt.% H and 20 wt.% Al MMMs. The curly brackets display the approximate periodicity of the alignment.

Alignment and clustering of m–ZIF−8 in the MMMs is further substantiated by analyzing the particle count of Zn (Figure 9).

In Figure 9, the vertical particle count of the respective MMM is visualized by the graph which overlays the Zn EDS map. Moreover, the aligned MMMs are rotated such that the direction of alignment, as indicated by the arrows in Figure 8, is positioned vertically. Both the 10 and 20 wt.% H MMMs show that the particle count throughout the membranes is evenly distributed, since the particle count graphs are approximately horizontal. On the other hand, clear peaks are present in the particle graphs of the 10 and 20 wt.% Al MMMs that occur periodically (indicated by the curly brackets in Figure 9). As stated previously, the aligned pathways and enriched regions in the 10 wt.% Al MMM are narrower and smaller than the 20 wt.% Al MMM, which is visualized by the smaller periodicity of the 10 wt.% MMM (i.e., the smaller curly brackets). Lastly, the peaks of the particle count graphs are more delicately pronounced in the 10 wt.% Al MMM than in the 20 wt.% Al MMM, which is attributed to the increased brittleness of the locally m–ZIF−8 aligned pathway. As an increasing MOF content in MMMs results in an increased brittleness, it is more difficult to keep an aligned m–ZIF−8 path intact when the sample is cryogenically fractured for the SEM/EDS analysis [30,44].

**Figure 10:**
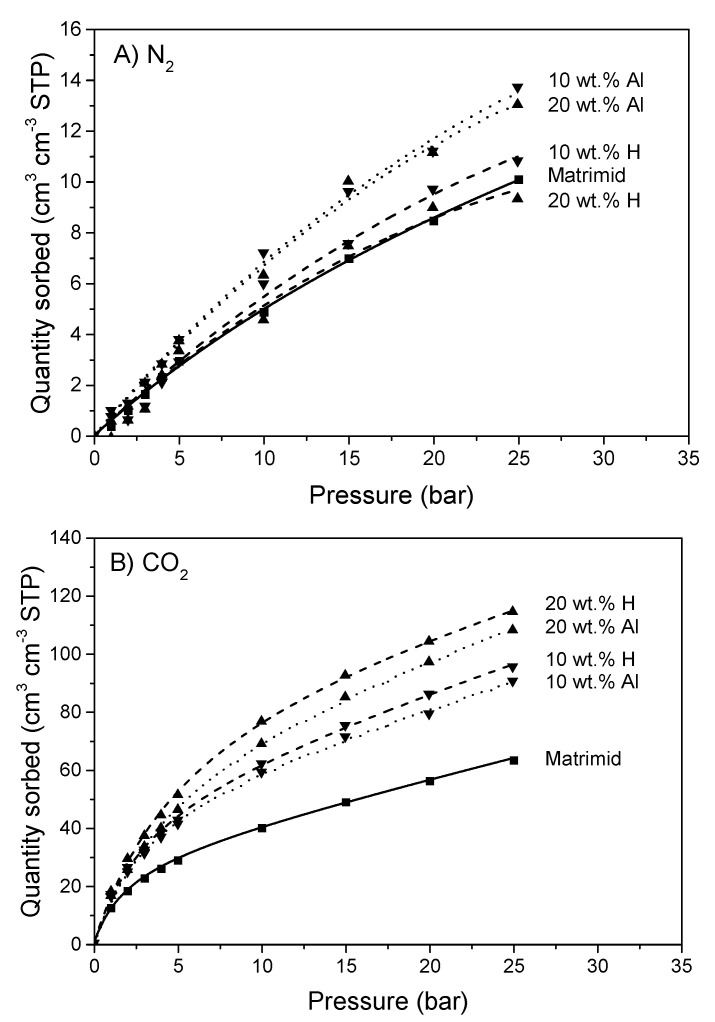
(**A**) N_2_ and (**B**) CO_2_ sorption of Matrimid, 10 wt.% H, 10 wt.% Al, 20 wt.% H and 20 wt.% Al MMMs.

N_2_ and CO_2_ sorption isotherms of Matrimid and the MMMs are shown in Figure 10.

The sorption of N_2_ in Matrimid and the MMMs exhibits predominantly Henry sorption behavior. It is notable that the aligned MMMs have an increased sorption of N_2_, with respect to both Matrimid and the homogeneous MMMs. Since m–ZIF−8 (Figure 6) has a higher N_2_ uptake than Matrimid (Figure 10), it is expected that all MMMs should have an increased N_2_ uptake. However, this is only observed for the aligned MMMs, which means that the N_2_ sorption sites of m–ZIF−8 in the aligned MMMs are more readily accessible than in the homogeneous MMMs. Therefore, it is concluded that ZIF−8 alignment and local enrichment effectively decrease the polymer content between the MOF particles. The uptake of CO_2_ in Matrimid and the MMMs occurs according to the dual-mode sorption model (i.e., Henry and Langmuir sorption. The aligned MMMs show a lower CO_2_ sorption than the homogeneous MMMs at elevated pressures. This decrease in solubility is ascribed to increased CO_2_ uptake at the ZIF/matrix interface, since it was demonstrated that at elevated CO_2_ pressure (starting at 10 bar) matrix derigidification at the interface causes an increased CO_2_ uptake [45]. The aligned and enriched MMMs have a decreased polymer content between the MOF particles, causing a decreased CO_2_ induced rigidification at elevated pressures. This results in a slightly lowered CO_2_ sorption relative to the homogeneous MMM. Increasing the m–ZIF−8 content in the MMMs results in a higher CO_2_ sorption, due to the high CO_2_ sorption capacity of m–ZIF−8 (Figure 6) in comparison with Matrimid (Figure 10).

**Figure 11:**
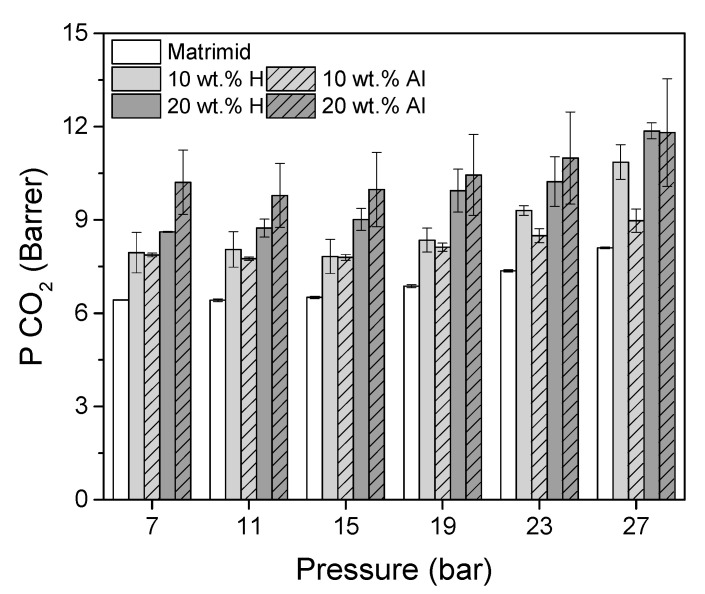
Single gas CO_2_ permeability (P_CO_2__) of Matrimid, 10 wt.% H, 10 wt.% Al, 20 wt.% H and 20 wt.% Al MMMs as function of pressure.

The influence of m–ZIF−8 content in the MMMs and effect of alignment on the CO_2_ single gas permeability is shown in Figure 11.

The CO_2_ permeability of Matrimid increases from 6.4 Barrer at 11 bar to 8.1 Barrer at 27 bar once its plasticization pressure is reached, which lies between 10 and 12 bar CO_2_ pressure [46]. The Matrimid CO_2_ permeability is slightly lower than values reported elsewhere in literature [29,44,46]. This observation is attributed to the difference in annealing protocol, which strongly influences the permeability [29]. The incorporation of 10 wt.% m–ZIF−8 results in an increased CO_2_ permeability of the homogeneous and aligned MMMs relative to pure Matrimid. Counterintuitively, since pure ZIF−8 has a high CO_2_ permeability, this increased CO_2_ permeability is caused by an increased CO_2_ solubility, while the CO_2_ diffusivity decreases (Table 1) [25]. This decrease in diffusivity is ascribed to matrix rigidification around the MOF particles [46,47]. Alignment at 10 wt.% m–ZIF−8 content does not influence the CO_2_ permeability, with respect to the homogeneous MMM. The CO_2_ permeability of both 10 wt.% MMMs starts to increase from 15 bar CO_2_ feed pressure, i.e., plasticization occurs. This increased plasticization pressure in MOF/Matrimid MMMs is in agreement with literature [46].

Further increasing the m–ZIF−8 content resulted in an increased CO_2_ permeability. For both the 20 wt.% H and Al MMMs, a further increase in CO_2_ solubility is observed (Table 1). However, in contrast with the 10 wt.% MMMs, a clear difference in diffusivity is visible for the 20 wt.% MMMs. The diffusivity of the 20 wt.% H MMM is decreased with respect to the 10 wt.% H MMM, which can be attributed to further matrix rigidification. On the other hand, the diffusivity of the 20 wt.% Al MMM increased, with respect to the 10 wt.% Al MMM. Even though the alignment direction in the 20 wt.% Al MMM was not perfectly parallel to the permeation path, it can be concluded that alignment and local MOF enrichment successfully increased the CO_2_ diffusivity and subsequently the permeability of the 20 wt.% MMM, with respect to the 20 wt.% H MMM. A maximum increase of 19% in CO_2_ permeability was observed at 7 bar, which indicates that alignment is predominantly beneficial below the plasticization temperature. Thus, the performance of MMMs can be improved by controlling the spatial distribution of the MOF additive.

**Figure 12:**
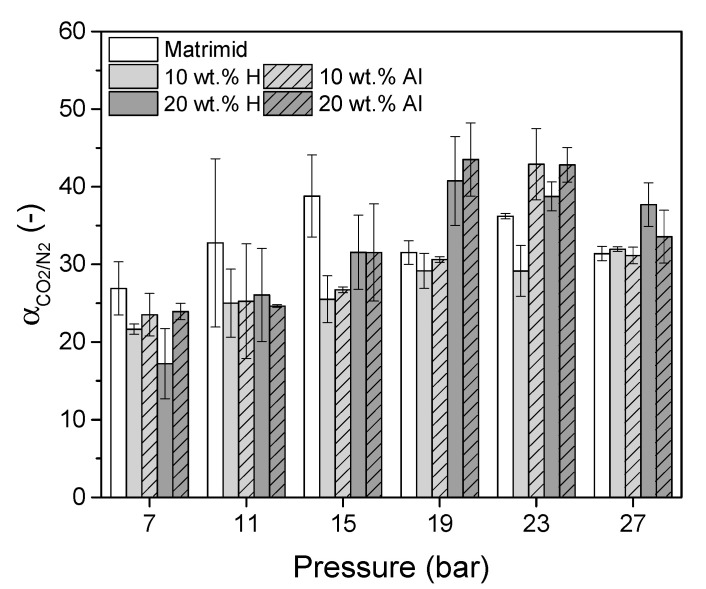
Ideal CO_2_/N_2_ selectivity of Matrimid, 10 wt.% H, 10 wt.% Al, 20 wt.% H and 20 wt.% Al MMMs as function of pressure.

The ideal CO_2_/N_2_ selectivity of Matrimid, 10 wt.% H, 10 wt.% Al, 20 wt.% H and 20 wt.% Al MMMs as function of pressure are shown in Figure 12. 

At low feed pressure, pure Matrimid has a higher ideal selectivity than the MMMs. This means that m–ZIF−8 enhances next to the CO_2_ permeability (Figure 11) also the N_2_ permeability, but the relative increase in N_2_ permeability is higher than the relative increase in CO_2_ permeability. Since single crystal ZIF−8 has a selectivity which is at maximum equal to the selectivity of Matrimid, it can be concluded that non idealities between the matrix-MOF interface are present [25]. Nonetheless, aligning m–ZIF−8 has a positive response on the permeability without compromising the ideal selectivity up to 15 bar, with respect to the homogeneous MMMs. The facts that overall, no significant differences are present between the ideal selectivity of the homogeneous and aligned MMMs (Figure 12) and that the solubility of N_2_ is higher in the aligned MMMs than in the homogeneous MMMs (Figure 10) prove that the aligned MMMs enhance the CO_2_ diffusivity more than the N_2_ diffusivity, relative to the homogeneous MMMs. It is argued that this enhanced CO_2_ diffusivity is caused by the different kinetic diameters of CO_2_ (3.30 Å) and N_2_ (3.64 Å) and the effect of the aligned and enriched ZIF−8 pathways. Since the pore aperture of ZIF−8 is 3.4 Å, CO_2_ (with a kinetic diameter smaller than the ZIF−8 pore aperture) will diffuse more easily through the MOF than N_2_ (with a kinetic diameter bigger than the ZIF−8 pore aperture). Moreover, the increased ZIF−8 interconnectivity in the aligned MMMs causes preferential permeation through the MOF relative to the matrix, since the permeability of pure ZIF−8 is significantly higher than the permeability of Matrimid. As a result, the CO_2_ diffusivity will thus relatively be promoted more than the N_2_ diffusivity in the aligned MMMs than in the homogeneous MMMs. With increasing CO_2_ pressure, the effect of plasticization on the selectivity is observed. Due to the plasticization induced increased CO_2_ permeability, the ideal selectivity of all membranes increase (an opposite trend is expected for mixed gas permeation experiments above the plasticization pressure). Clearly, Matrimid plasticizes most pronounced at 11 to 15 bar feed pressure, while the MMM start to plasticize at 15 bar feed pressure.

## 4. Conclusions

ZIF−8 was successfully magnetized by the incorporation of Fe_3_O_4_. The presence of Fe_3_O_4_ in m–ZIF−8 showed a decrease in BET surface area and CO_2_ and N_2_ solubility, with respect to pure ZIF−8. Alignment of m–ZIF−8 in Matrimid was visually present by SEM-EDS analysis and showed clear m–ZIF−8 enriched pathways and regions in the aligned and clustered MMMs both at 10 and 20 wt.%. Due to the limitation of a permanent magnetic field lines, it was observed that the alignment was not fully perpendicular to the membrane surface, which increased the effective length of the m–ZIF−8 pathway through the MMM. At 20 wt.% m–ZIF−8 incorporation in Matrimid, alignment was proven to increase the CO_2_ permeability at maximum with 19% at 7 bar, while no CO_2_/N_2_ selectivity loss of was observed, with respect to the homogeneous 20 wt.% MMM. Therefore, it was concluded that at 20 wt.% m–ZIF−8 content, the alignment results in sufficiently m–ZIF−8 enriched aligned clusters throughout the MMM matrix that established an increase in diffusivity and subsequently permeability. The use of a homogeneous magnetic field will straighten the percolation path and will result in a further improved membrane performance at even lower weight percentages of magnetic MOFs. Nonetheless, this paper shows that alignment of MOF particles throughout the matrix creating permeation highways is beneficial to enhance the CO_2_ permeability without adversely affecting the selectivity.

## Figures and Tables

**Table 1:** CO_2_ Permeability (P), solubility coefficient (S) and diffusion coefficient (D) of Matrimid, 10 wt.% H, 10 wt.% Al, 20 wt.% H and 20 wt.% Al MMMs at 11 bar.

Membrane	P [Barrer]	S [cm^3^ STP/(cm^3^ · cmHg)]	D [10^−8^ cm^2^/s]
Matrimid	6.4	0.051	1.3
10 wt.% H	8.1	0.078	1.0
10 wt.% Al	7.8	0.074	1.0
20 wt.% H	8.7	0.096	0.9
20 wt.% Al	9.8	0.087	1.1

## References

[1] Ismail A.F., Khulbe K.C., Matsuura T. (2015). Gas Separation Membranes: Polymeric and Inorganic.

[2] Robeson L.M. (1991). Correlation of separation factor versus permeability for polymeric membranes. J. Memb. Sci..

[3] Robeson L.M. (2008). The upper bound revisited. J. Memb. Sci..

[4] Biswal B.P., Chaudhari H.D., Banerjee R., Kharul U.K. (2016). Chemically Stable Covalent Organic Framework (COF)-Polybenzimidazole Hybrid Membranes: Enhanced Gas Separation through Pore Modulation. Chem. Eur. J..

[5] Thür R., Van Velthoven N., Slootmaekers S., Didden J., Verbeke R., Smolders S., Dickmann M., Egger W., De Vos D., Vankelecom I.F.J. (2019). Bipyridine-based UiO−67 as novel filler in mixed-matrix membranes for CO_2_-selective gas separation. J. Memb. Sci..

[6] Kertik A., Wee L.H., Pfannmöller M., Bals S., Martens J.A., Vankelecom I.F.J. (2017). Highly selective gas separation membrane using in situ amorphised metal-organic frameworks. Energy Environ. Sci..

[7] Yuan J., Zhu H., Sun J., Mao Y., Liu G., Jin W. (2017). Novel ZIF−300 Mixed-Matrix Membranes for Efficient CO_2_ Capture. ACS Appl. Mater. Interfaces.

[8] Jeazet H.B.T., Staudt C., Janiak C. (2012). Metal–organic frameworks in mixed-matrix membranes for gas separation. Dalt. Trans..

[9] Li H., Wang K., Sun Y., Lollar C.T., Li J., Zhou H.C. (2018). Recent advances in gas storage and separation using metal–organic frameworks. Mater. Today.

[10] Park K.S., Ni Z., Côté A.P., Choi J.Y., Huang R., Uribe-Romo F.J., Chae H.K., O’Keeffe M., Yaghi O.M. (2006). Exceptional chemical and thermal stability of zeolitic imidazolate frameworks. Proc. Natl. Acad. Sci. USA.

[11] Yuan S., Feng L., Wang K., Pang J., Bosch M., Lollar C., Sun Y., Qin J., Yang X., Zhang P. (2018). Stable Metal-Organic Frameworks: Design, Synthesis, and Applications. Adv. Mater..

[12] Phan A., Doonan C.J., Uribe-Romo F.J., Knobler C.B., O’Keeffe M., Yaghi O.M. (2010). Synthesis, Structure, and Carbon Dioxide Capture Properties of Zeolitic Imidazolate Frameworks. Acc. Chem. Res..

[13] Chen B., Yang Z., Zhu Y., Xia Y. (2014). Zeolitic imidazolate framework materials: Recent progress in synthesis and applications. J. Mater. Chem. A.

[14] Pan Y., Liu Y., Zeng G., Zhao L., Lai Z. (2011). Rapid synthesis of zeolitic imidazolate framework−8 (ZIF−8) nanocrystals in an aqueous system. Chem. Commun..

[15] Tanaka S., Kida K., Okita M., Ito Y., Miyake Y. (2012). Size-Controlled synthesis of zeolitic imidazolate framework−8 (ZIF−8) crystals in an aqueous system at room temperature. Chem. Lett..

[16] Cravillon J., Nayuk R., Springer S., Feldhoff A., Huber K., Wiebcke M. (2011). Controlling Zeolitic Imidazolate Framework Nano- and Microcrystal Formation: Insight into Crystal Growth by Time-Resolved In Situ Static Light Scattering. Chem. Mater..

[17] Lee Y.R., Jang M.S., Cho H.Y., Kwon H.J., Kim S., Ahn W.S. (2015). ZIF−8: A comparison of synthesis methods. Chem. Eng. J..

[18] Bustamante E.L., Fernández J.L., Zamaro J.M. (2014). Influence of the solvent in the synthesis of zeolitic imidazolate framework−8 (ZIF−8) nanocrystals at room temperature. J. Colloid Interface Sci..

[19] Lu G., Li S., Guo Z., Farha O.K., Hauser B.G., Qi X., Wang Y., Wang X., Han S., Liu X. (2012). Imparting functionality to a metal-organic framework material by controlled nanoparticle encapsulation. Nat. Chem..

[20] Zhang T., Zhang X., Yan X., Kong L., Zhang G., Liu H., Qiu J., Yeung K.L. (2013). Synthesis of Fe_3_O_4_@ZIF−8 magnetic core-shell microspheres and their potential application in a capillary microreactor. Chem. Eng. J..

[21] Jiang X., Chen H.Y., Liu L.L., Qiu L.G., Jiang X. (2015). Fe_3_O_4_ embedded ZIF−8 nanocrystals with ultra-high adsorption capacity towards hydroquinone. J. Alloy. Compd..

[22] Venna S.R., Carreon M.A. (2015). Metal organic framework membranes for carbon dioxide separation. Chem. Eng. Sci..

[23] Guan W., Dai Y., Dong C., Yang X., Xi Y. (2020). Zeolite imidazolate framework (ZIF)-based mixed matrix membranes for CO 2 separation: A review. J. Appl. Polym. Sci..

[24] Pimentel B.R., Parulkar A., Zhou E., Brunelli N.A. (2014). Zeolitic Imidazolate Frameworks: Next-Generation Materials for Energy-Efficient Gas Separations. ChemSusChem.

[25] Chen C., Ozcan A., Yazaydin A.O., Ladewig B.P. (2019). Gas permeation through single-crystal ZIF−8 membranes. J. Memb. Sci..

[26] Shahid S., Nijmeijer K., Nehache S., Vankelecom I., Deratani A., Quemener D. (2015). MOF-mixed matrix membranes: Precise dispersion of MOF particles with better compatibility via a particle fusion approach for enhanced gas separation properties. J. Memb. Sci..

[27] Wang Z., Wang D., Zhang S., Hu L., Jin J. (2016). Interfacial Design of Mixed Matrix Membranes for Improved Gas Separation Performance. Adv. Mater..

[28] Sánchez-Laínez J., Zornoza B., Friebe S., Caro J., Cao S., Sabetghadam A., Seoane B., Gascon J., Kapteijn F., Le Guillouzer C. (2016). Influence of ZIF−8 particle size in the performance of polybenzimidazole mixed matrix membranes for pre-combustion CO2 capture and its validation through interlaboratory test. J. Memb. Sci..

[29] Song Q., Nataraj S.K., Roussenova M.V., Tan J.C., Hughes D.J., Li W., Bourgoin P., Alam M.A., Cheetham A.K., Al-Muhtaseb S.A. (2012). Zeolitic imidazolate framework (ZIF−8) based polymer nanocomposite membranes for gas separation. Energy Environ. Sci..

[30] Mahdi E.M., Tan J.C. (2016). Mixed-matrix membranes of zeolitic imidazolate framework (ZIF−8)/Matrimid nanocomposite: Thermo-mechanical stability and viscoelasticity underpinning membrane separation performance. J. Memb. Sci..

[31] Cheng F., Marshall E.S., Young A.J., Robinson P.J., Bouillard J.S.G., Adawi A.M., Vermeulen N.A., Farha O.K., Reithofer M.R., Chin J.M. (2017). Magnetic Control of MOF Crystal Orientation and Alignment. Chem. Eur. J..

[32] Sharma A., Tripathi B., Vijay Y.K. (2010). Dramatic Improvement in properties of magnetically aligned CNT/polymer nanocomposites. J. Memb. Sci..

[33] Sharma A., Kumar S., Tripathi B., Singh M., Vijay Y.K. (2009). Aligned CNT/Polymer nanocomposite membranes for hydrogen separation. Int. J. Hydrog. Energy.

[34] Qiao Z., Zhao S., Wang J., Wang S., Wang Z., Guiver M.D. (2016). A Highly Permeable Aligned Montmorillonite Mixed-Matrix Membrane for CO_2_ Separation. Angew. Chem..

[35] Jung H.S., Kwon S.H., Choi H.J., Jung J.H., Kim Y.G. (2016). Magnetic carbonyl iron/natural rubber composite elastomer and its magnetorheology. Compos. Struct..

[36] Yao J., Sun Y., Wang Y., Fu Q., Xiong Z., Liu Y. (2018). Magnet-induced aligning magnetorheological elastomer based on ultra-soft matrix. Compos. Sci. Technol..

[37] Weber R., Orsino S., Lallemant N., Verlaan A. (2000). Combustion of natural gas with high-temperature air and large quantities of flue gas. Proc. Combust. Inst..

[38] Ibbs T.L., Underwood L. (1926). A comparison of the behaviour in thermal diffusion of nitrogen and carbon monoxide, and of nitrous oxide and carbon dioxide. Proc. Phys. Soc..

[39] Bongers W., Bouwmeester H., Wolf B., Peeters F., Welzel S., van den Bekerom D., den Harder N., Goede A., Graswinckel M., Groen P.W. (2017). Plasma-driven dissociation of CO_2_ for fuel synthesis. Plasma Process. Polym..

[40] Petcharoen K., Sirivat A. (2012). Synthesis and characterization of magnetite nanoparticles via the chemical co-precipitation method. Mater. Sci. Eng. B.

[41] Nordin N.A.H.M., Ismail A.F., Mustafa A., Murali R.S., Matsuura T. (2014). The impact of ZIF−8 particle size and heat treatment on CO_2_/CH_4_ separation using asymmetric mixed matrix membrane. RSC Adv..

[42] Venkatasubramanian A., Navaei M., Bagnall K.R., McCarley K.C., Nair S., Hesketh P.J. (2012). Gas Adsorption Characteristics of Metal-Organic Frameworks via Quartz Crystal Microbalance Techniques. J. Phys. Chem. C.

[43] Chokbunpiam T., Fritzsche S., Chmelik C., Caro J., Janke W., Hannongbua S. (2016). Gate Opening, Diffusion, and Adsorption of CO_2_ and N_2_ Mixtures in ZIF−8. J. Phys. Chem. C.

[44] Ordoñez M.J.C., Balkus K.J., Ferraris J.P., Musselman I.H. (2010). Molecular sieving realized with ZIF−8/Matrimid^®^ mixed-matrix membranes. J. Memb. Sci..

[45] Balçık M., Tantekin-Ersolmaz S.B., Ahunbay M.G. (2020). Interfacial analysis of mixed-matrix membranes under exposure to high-pressure CO2. J. Memb. Sci..

[46] Shahid S., Nijmeijer K. (2014). Performance and plasticization behavior of polymer-MOF membranes for gas separation at elevated pressures. J. Memb. Sci..

[47] Shahid S., Nijmeijer K. (2014). High pressure gas separation performance of mixed-matrix polymer membranes containing mesoporous Fe(BTC). J. Memb. Sci..

